# Life History Traits and Fishery Dynamics of Speckled Shrimp, *Metapenaeus monoceros* (Fabricius, 1798), Along the Saudi Arabian Red Sea Coast

**DOI:** 10.3390/biology14040406

**Published:** 2025-04-11

**Authors:** Sheeja Gireesh, Eyüp Mümtaz Tıraşın, Goutham Bharathi Muthu Palani, Santhosh Kumar Charles, Sirajudheen Thayyil Kadengal, Ronald Grech Santucci, Ricardo Clapis Garla, Zahra Okba, Adel M. S. Adam, Mark Dimech

**Affiliations:** 1 KAUST Beacon Development Department, National Transformation Institute, King Abdullah University of Science and Technology, Thuwal 23955-6900, Saudi Arabia; sheeja.gireesh@kaust.edu.sa (S.G.); goutham.muthupalani@kaust.edu.sa (G.B.M.P.); santhosh.charles@kaust.edu.sa (S.K.C.); sirajudheen.kadengal@kaust.edu.sa (S.T.K.); ronald.grechsantucci@kaust.edu.sa (R.G.S.); ricardo.garla@kaust.edu.sa (R.C.G.); zahra.okba@kaust.edu.sa (Z.O.); adel.adam@kaust.edu.sa (A.M.S.A.); mark.dimech@kaust.edu.sa (M.D.); 2 Institute of Marine Sciences and Technology, Dokuz Eylül University, İnciraltı, İzmir 35340, Türkiye

**Keywords:** crustacean fisheries, shrimp trawling, Penaeidae, growth, mortality, maturity, stock assessment, spawning potential ratio, overfishing

## Abstract

*Metapenaeus monoceros* is the second most abundant commercially important shrimp species caught along the Saudi Arabian Red Sea coast. However, knowledge of its life history and population dynamics in the region remains limited, posing challenges for sustainable management. This study assessed the growth, maturity, and mortality of *M. monoceros* based on fishery-independent samples collected from shrimp trawling grounds off Al Qunfudhah and Jizan (October 2022–September 2023). Altogether, 10,859 specimens were examined, with a carapace length (*CL*) ranging from 7.98 to 49.10 mm and a total weight (*W*) in the range of 0.41 to 41.28 g. The observed sex ratio was 1.56:1 in favor of females. The *CL*–*W* relationship was *W* = 0.0024∙*CL*^2.58^. The seasonalized von Bertalanffy growth parameters were *CL*_∞_ = 50.66 mm, *K* = 0.35 year^−1^, *t_anchor_* = 0.43 years, *C* = 0.64, and *t_s_* = 0.61 years for females, and *CL*_∞_ = 38.97 mm, *K* = 0.55 year^−1^, *t_anchor_* = 0.51 years, *C* = 0.84, and *t_s_* = 0.39 years for males. Females reached their first sexual maturity at 21.12 mm *CL*. The exploitation rates (0.60 for both sexes) indicated overfishing. Spawning potential ratio analysis showed that the current fishing pressure reduces the spawning stock biomass to 23% of unexploited levels. A 50% reduction in fishing effort, extended seasonal closures, and selective gear modifications are recommended to rebuild the stock and ensure sustainability.

## Simple Summary

Marine shrimp fisheries primarily target penaeid species, which are in high demand in the global market. The considerable ecological and commercial importance of these species highlights the need for effective stock assessments to support appropriate management strategies. This study examines key population parameters, including growth, mortality, maturity, and morphometric relationships, to assess the status of the speckled shrimp *Metapenaeus monoceros* stock along the Saudi Arabian Red Sea coast. Growth parameter estimates indicate that females attain a larger asymptotic carapace length than males but grow at a slower rate. The exploitation rate for both sexes exceeds the optimum level, raising concerns regarding the sustainability of the fishery. The high proportion of immature individuals in the catch, combined with elevated fishing mortality and exploitation rates, strongly suggests that overfishing is occurring in the region. To ensure the long-term sustainability of this commercially valuable species, continued monitoring and the implementation of effective management measures are recommended.

## 1. Introduction

The speckled shrimp *Metapenaeus monoceros* (Fabricius, 1798) is a widely distributed penaeid species with a remarkable geographical range spanning the Indo-West Pacific region. Its native distribution encompasses a vast area, including the east coast of Africa, Madagascar, Tanzania, the Red Sea, the entire Indian coastal belt, Sri Lanka, Pakistan, Malaysia, the Straits of Malacca, Indonesia, Australia, and Japan [1,2,3]. Notably, *M. monoceros* has successfully migrated through the Suez Canal, establishing itself as a Lessepsian migrant in the eastern Mediterranean Sea [4]. This expansion has resulted in established populations along the coasts of Egypt, Israel, Syria, Lebanon, Turkey, Greece, and Tunisia [5,6,7,8]. Economically, the species plays a crucial role in regional fisheries, particularly in Bangladesh, where it constitutes more than 50% of the total shrimp landings [9]. This commercial importance underscores the species’ significance not only from an ecological perspective but also as a critical resource for local fishing communities and national seafood industries.

*M. monoceros* inhabits marine and brackish water environments, typically ranging from 10 to 30 m in depth, with occasional observations extending to a maximum recorded depth of 170 m [3,10]. The species demonstrates a strong preference for sandy and muddy habitats, which provide essential ecological niches for feeding, reproduction, and shelter. The species exhibits remarkable sexual dimorphism, with males attaining a maximum total length (*TL*) of 199 mm and females reaching 226 mm [11]. The species displays a relatively short life cycle, typically spanning 2–3 years [12,13]. Growth patterns also differ between sexes; males are heavier than females up to 77 mm *TL*, after which females exhibit greater weight due to maturation processes [14]. Furthermore, females are generally more abundant than males in the population [11,15,16,17]. Reproduction in *M. monoceros* is characterized by continuous spawning throughout the year, punctuated by seasonal peaks [18,19,20,21,22].

The fishery biology and stock characteristics of *M. monoceros* have been extensively studied in India [13,15,23,24,25]. Notably, trawl fisheries have led to overfishing of this species in both the Arabian Sea and the Bay of Bengal. Specifically, overfishing occurred along the Karwar, Veraval, and Mumbai coasts in the Arabian Sea [13,25]. Moreover, Nandakumar and Srinath [15] reported overfishing off the Kerala coast in the same region. Similarly, trawling has resulted in the overfishing of *M. monoceros* along the Kakinada and Visakhapatnam coasts in the Bay of Bengal [13,25]. The species is also targeted by trawling in the eastern Mediterranean, according to records for the southern coast of Turkey, and the continental shelf off Palestine, Egypt, and Tunisia [5,6,26,27,28]. Furthermore, overfishing has been documented in the industrial fishing zone of Bangladesh [29].

The shrimp fishery along the southeastern Red Sea coast of Saudi Arabia is predominantly based on *Penaeus semisulcatus* and *M. monoceros* [30]. Statistics from the Saudi Arabian Ministry of Environment, Water, and Agriculture (MEWA) indicate that annual shrimp landings along the Saudi Arabian Red Sea coast between 2017 and 2023 (reported collectively for all commercial shrimp species) ranged from 500 to 835 tonnes, with an average of 620 tonnes [31]. Despite the commercial and ecological importance of these species, scientific literature documenting their stock assessments along the Saudi Arabian Red Sea coast remains remarkably limited, creating a substantial knowledge gap in regards to regional marine resource management. Understanding the life history traits, growth and mortality parameters, and reproductive capacity of a species under current fishing pressure is essential for evaluating its stock status and ensuring sustainable management. This study aims to address this critical knowledge gap by investigating the growth, mortality, and reproduction of *M. monoceros* and conducting a preliminary assessment of stock status along the southeastern Red Sea coast. The findings of this study are expected to provide essential information for the sustainable management of this commercially important species.

## 2. Materials and Methods

### 2.1. Study Area

**Figure 1:**
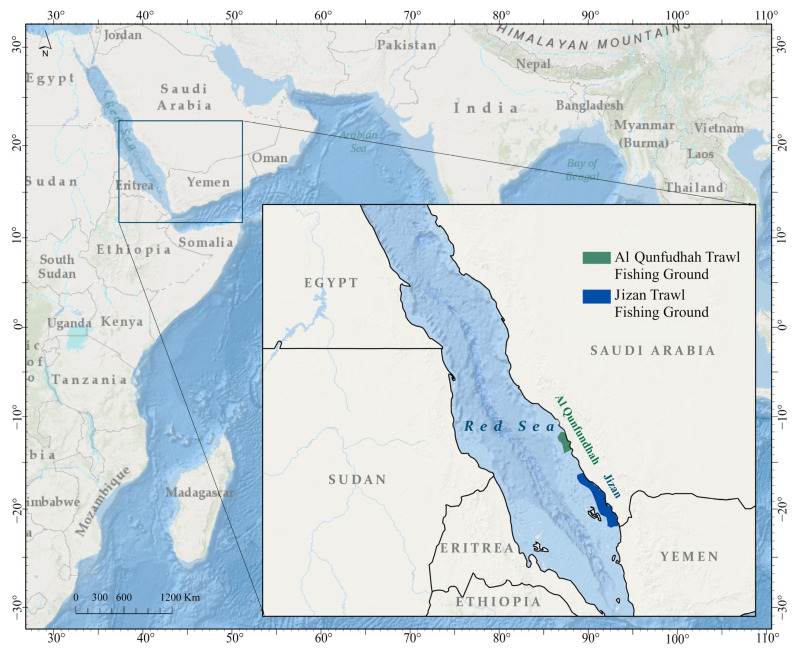
Locations of Al Qunfudhah and Jizan trawl fishing grounds along the southeastern Red Sea coast of Saudi Arabia.

Al Qunfudhah and Jizan are the only ports along the Red Sea coast of Saudi Arabia where shrimp trawl fisheries are legally permitted (Figure 1). Shrimp trawling in these areas is conducted exclusively by vessels registered as industrial vessels. These vessels are typically constructed from wood (often reinforced with fiberglass) or sheet metal [32]. According to national regulations [33], industrial vessels must be equipped with modern instrumentation, including echosounders, GPS, and communication systems, as well as safety equipment, refrigeration facilities, and winches. The length overall and main engine power of these vessels must not exceed 20 m and 250 HP, respectively. The MEWA retains the authority to issue or renew licenses, thereby regulating the number of operational fishing vessels and determining ownership, including for investor fishers who may not be directly involved in fishing activities. Additional input controls imposed by Saudi Arabia on the shrimp fishery include a seasonal closure between April and August, restricting trawling to designated fishing grounds, and prohibiting fishing within two nautical miles of the coastline or islands. The only technical measure currently in place is a regulation that prohibits the use of codend mesh sizes smaller than 38 mm [33].

Jizan, situated near the Farasan Islands, is the largest and most important fishing port along the Red Sea coast in terms of fleet size and fish production (Figure 1). The industrial fishing fleet registered in Jizan comprises 151 vessels, whereas only 24 vessels are registered in Al Qunfudhah [32,34]. In Jizan, trawling is typically conducted at an acute angle to the coastline, with fishing depths ranging from 10 to 40 m. In contrast, in Al Qunfudhah, trawling operations are primarily conducted parallel to the coastline, with fishing grounds ranging in depth from 10 to 30 m. The trawling grounds in both areas are primarily sandy, interspersed with patches of muddy substrate [34,35]. In addition to trawling, industrial vessels may also engage in purse seining, depending on the seasonal closure of the shrimp trawl fishery [32].

### 2.2. Biological Data Collection

Monthly biological sampling was carried out off the coasts of Al Qunfudhah and Jizan, along the southeastern Red Sea (Figure 1), from October 2022 to September 2023. For experimental trawl surveys, commercial shrimp trawlers registered at these ports were chartered. The trawl hauls in these surveys closely replicated commercial fishing operations, maintaining consistency in trawl net specifications, fishing locations, haul duration, towing speed, and other operational parameters. All hauls were conducted at night at an average towing speed of three knots, with each tow lasting approximately three hours. The number of hauls varied from month to month due to weather conditions, but an average of nine hauls per month were conducted at each location. Over the course of the study, a total of 110 hauls were completed in Al Qunfudhah and 108 in Jizan. Every haul utilized the standard 40 mm diamond mesh codend, which is commonly used in commercial shrimp trawling operations in the region [32,35]. A special permit was obtained from the MEWA to continue experimental trawl surveys during the closed season between April and August.

The primary target species for shrimp trawlers operating within Saudi Arabian territorial waters in the Red Sea include *P. semisulcatus*, *M. monoceros*, *P. pulchricaudatus*, *P. indicus*, and *P. hathor* [30,34]. After the trawl was brought onboard, all target shrimp species were separated from the rest of the catch. Shrimp from each haul, regardless of species, were stored separately on ice in separate containers and transported to the laboratory for detailed processing. In the laboratory, specimens were first sorted by species. All *M. monoceros* individuals were processed, and various biological variables were registered. Carapace length (*CL*) was measured to the nearest 0.01 mm using a digital caliper, and total weight (*W*) was recorded at the nearest 0.01 g for each specimen. Sex was determined macroscopically based on the presence of petasma in males or thelycum in females [36].

Gonadal maturity stages were determined only in female shrimp using a modified methodology described by Amanat and Qureshi [37] and Abdallah et al. [27]. Ovarian maturation stages were classified into four distinct stages, based on coloration and size of the ovaries—(Stage I) Immature or undeveloped: ovaries are thin, translucent, and underdeveloped, with poorly defined anterior and lateral lobes; (Stage II) Developing: ovaries appear creamy white to yellowish and are larger in size, and in more advanced cases within this stage, they occupy most of the dorsal portion and range from yellow to light green in color; (Stage III) Mature: ovaries are fully developed, occupying the entire dorsal region, are dark green in color, and are visible through the exoskeleton; (Stage IV) Spent: ovaries resemble those in Stage II in color but are more enlarged, flaccid in texture, and reduced in turgidity, indicating post-spawning condition (Figure S1).

### 2.3. Length–Weight Relationship

Estimated mean values of *CL* and *W* measurements by sex were compared using independent *t*-tests. Prior to these analyses, the assumptions of normality and homoscedasticity were evaluated using *F*-tests, Shapiro–Wilk normality tests, and normal quantile-quantile plots [38]. If the normality assumption was met but variances were heteroscedastic, Welch’s approximate *t*-test was applied. For data that violated both assumptions, a log transformation was attempted. If this transformation did not resolve the issues, the non-parametric Mann–Whitney test was employed [38]. The relationship between *CL* and *W* was examined using linear regression analysis after log-transforming all data pairs, based on the assumption of a multiplicative error structure [39].

(1) $$W=a\cdot {CL}^{b}$$

In this nonlinear relationship, *a* represents the coefficient for the ratio between *W* and *CL*, while *b* is the exponent describing how the body mass (*W*) of a shrimp changes as its size (*CL*) increases. An analysis of covariance (ANCOVA) [38] was conducted to determine whether the pairs of *a* and *b* values differed between female and male shrimp. Departures from isometric growth, i.e., whether the estimated *b* significantly deviated from the hypothesized value of 3, were assessed by determining whether the confidence intervals for each *b* comprised 3 or not [39,40].

### 2.4. Size at First Maturity

The median size at first sexual maturity, i.e., the *CL* at which 50% of speckled shrimp in the population reach sexual maturity (*CL*_50_), was estimated exclusively for females using logistic regression analysis [41], as described by Aydın and Tıraşın [42]. The maturity status of each female shrimp was classified as binary data (Stage I was deemed “immature = 0”, while Stage II and above were designated as “mature = 1”). The logistic model was fitted to these binomial maturity data. To evaluate the uncertainty of the *CL*_50_ estimate, the bootstrap method [42,43] was applied, generating 5000 bootstrap samples. A nonparametric 95% confidence interval for *CL*_50_ was then estimated using the bias-corrected and accelerated (BCa) method [43].

### 2.5. Growth

The growth of *M. monoceros* in the southeastern Red Sea was described by means of the seasonally oscillating version of the von Bertalanffy growth model [44], as follows:

(2) $${CL}_{t}={CL}_{\infty }\cdot \left(1-e^{-K\cdot \left(t-t_{0}\right)-\left(\frac{C\cdot K}{2\pi }\right)\cdot \left(sin\left(2\pi \cdot \left(t-t_{s}\right)\right)-sin\left(2\pi \cdot \left(t_{0}-t_{s}\right)\right)\right)}\right)$$

where *CL_t_* is the expected *CL* at age *t* years, *CL*_∞_ is the asymptotic *CL*, *K* is the growth coefficient or curvature parameter indicating the rate at which shrimp grow towards their *CL*_∞_, *t*_0_ is the theoretical age at 0 mm *CL*, *C* is a constant which shows the amplitude of the growth oscillation, and *t_s_* is the fraction of a year defining the beginning of the sinusoidal growth oscillation with respect to *t*_0_, i.e., relative to the age of recruitment [44,45]. When *C* equals 0, then there is no seasonal oscillation, and the above model reduces to the traditional parameterization of the von Bertalanffy growth model created by Beverton and Holt [46].

The recently developed TropFishR package (version 1.6.4) [47,48], which incorporates the electronic length frequency analysis (ELEFAN) method [45,49], was utilized to estimate the parameters of the seasonally oscillating von Bertalanffy growth model for female and male shrimp separately, except for *t*_0_. The von Bertalanffy growth model requires actual age information (*t*) as an independent variable; however, length frequency samples only provide the time interval between samplings, making it impossible to estimate *t*_0_ solely with length frequency data [45]. Instead, TropFishR provides a related parameter, “*t_anchor_*”, which represents the fraction of the year ranging between 0 and 1, where the growth curve crosses 0 mm *CL* for a given cohort [47]. A powerful new optimization procedure in TropFishR, the ELEFAN_GA function, which simultaneously searches across all parameters, was employed using a moving average (MA) over five *CL* intervals. Using the ELEFAN_GA function offers a significant advantage over the basic ELEFAN, as it efficiently models the seasonally oscillating version of the von Bertalanffy growth model. Monthly *CL* distributions, grouped into 2 mm class intervals for each sex, were used as input data. A comparison of growth performances between females and males was conducted using the growth performance index (*φ*′) [50], as follows:

(3) $$\varphi ‘={log}_{10}\left(K\right)+2\cdot {log}_{10}({CL}_{\infty })$$

### 2.6. Mortality

The total mortality rate (*Z*) for each sex was estimated from the linearized length-converted catch curve analysis [51]. In accordance with the work of Quinn and Deriso [39], Cope and Hamel [52], and Maunder et al. [53], the natural mortality rate (*M*) was estimated for each sex using three different methods recommended by Maunder et al. [53] to enhance methodological robustness and reduce potential bias from relying on a single approach [54,55].

(4) M=5.4tmax

(5) M=1.55·K

(6) $$M=4.118\cdot K^{0.73}\cdot {CL}_{\infty }^{-0.33}$$

*t*_max_ in Equation (4) represents the maximum age, or longevity, of a species. Based on previous studies [12,13], *t_max_* was assumed to be 3 years for female shrimp and 2 years for males. *CL*_∞_ and *K* are the parameters of the von Bertalanffy growth model, as described earlier. The *M* value ultimately used for further analysis was determined by averaging the three independent estimates derived from the abovementioned methods. Since *Z* consists of both *M* and the fishing mortality rate (*F*), once *Z* and *M* were obtained, *F* was estimated using the following relationship:

(7) F=Z−M

### 2.7. Stock Status Evaluation

A preliminary evaluation of the stock status of *M. monoceros* in the southeastern Red Sea was conducted by estimating the current levels of the exploitation rate (*E*) and the spawning potential ratio (SPR). The *E* was calculated separately for each sex as the ratio of *F* to *Z*, as follows:

(8) $$E=\frac{F}{Z}$$

SPR is a key metric used to assess the reproductive potential of a stock under current fishing pressure, with a particular focus on females, as they are responsible for egg production, which drives recruitment. Goodyear [56] defined SPR as the equilibrium spawning stock biomass per recruit (SSBpR) at a given level of fishing mortality, divided by the equilibrium SSBpR in the absence of fishing (i.e., when only natural mortality affects the stock). SPR analysis is an extension of the prediction models, such as those developed by Thompson and Bell [51] or Beverton and Holt [46], which are traditionally used to estimate yield per recruit (YpR) and biomass per recruit (BpR). BpR represents the average biomass contributed by each recruit in the stock. The calculation of SSBpR for a fished stock is analogous to the computation of BpR; however, instead of considering the entire life span of a recruiting cohort, the focus is on the mature females within the cohort that are capable of spawning [56,57]. Like BpR, SPR has a maximum value of 1 when no fishing occurs (*F* = 0), and it declines towards 0 as *F* increases. SPR may also be expressed as a percentage, representing the proportion of the unfished SSBpR that remains under a given *F* value (e.g., at *F* = 0, %SPR is at 100%).

A length-based Thompson and Bell YpR model [51] was applied to estimate the SPR of female speckled shrimp. This analysis focused exclusively on females due to their direct contribution to egg production and consequently, their critical role in determining population reproductive capacity. The model required sex-specific growth and mortality parameters, including *CL_∞_*, *K*, and *M*, as well as the number of shrimp in each 2 mm *CL*-class (using the same size intervals and counts employed for the von Bertalanffy growth model parameter estimation). Additional model inputs included the mean weights by *CL*-class and the *CL* at first capture (*CL_c_*), which was set at 17 mm, based on findings from a recent study by Santucci et al. [35] conducted with commercial trawl boats and gear off Jizan. The proportion of mature females in each *CL*-class was predicted using logistic regression analysis. The YpR, BpR, and SSBpR were estimated as a function of varying *F* levels and expressed in mass (g).

Biological reference points (BRPs) derived from YpR models are expressed as *F* rates, which serve as indicators of stock status [39]. From the YpR model, two key BRPs are often derived: *F*_max_ and *F*_0.1_. *F*_max_; the *F* that maximizes YpR is considered a limit reference point (LRP) [58]. *F*_0.1_ is the *F* at which the slope of the YpR curve is reduced to 10% of its slope at the origin [40]. This reference point is often regarded as a precautionary target reference point (TRP) for sustainable fishery management [59]. The LRP defines a critical threshold that should not be breached to prevent serious harm to the stock, indicating an unsustainable biomass or fishing pressure level. In contrast, the TRP represents the desired level of *F* or stock biomass, aimed at achieving long-term sustainability and optimal yield while maintaining the stock’s health and productivity [58,59,60].

YpR analysis focuses primarily on assessing growth overfishing and does not account for the impact of fishing on the reproductive capacity of the stock [39,61,62]. Alternatively, SPR analysis offers critical information on recruitment overfishing by evaluating the effects of fishing on the stock’s ability to replenish itself through adequate SSB [39,63,64]. The minimum SPR value widely recommended as a TRP to safeguard against stock depletion and prevent recruitment overfishing is 0.4 [56,61,62]. The *F* associated with this TRP is commonly referred to as *F*_40%_. Although an SPR value as low as 0.2 has been proposed as an LRP [56,63], Walters and Martell [64] emphasized that historical stock-recruitment data show a considerably higher risk of recruitment overfishing when SPR falls below 0.3. Accordingly, an SPR of 0.3 is adopted as the LRP in this evaluation.

Additionally, in this study, an *E* value of 0.5 has been employed as an LRP [58,65], representing a threshold of sustainable fishing pressure that should ideally be avoided, to evaluate the status of speckled shrimp in the region.

All statistical tests were conducted with R software version 4.4.1 [66] with a significance level of 5%.

## 3. Results

### 3.1. Distribution of the Samples

A total of 103,758 individuals from six commercially valuable shrimp species were sampled from the fishing grounds in the southeastern Red Sea during the study period. The catch composition was dominated by *P. semisulcatus* (83%) and *M. monoceros* (10%). *P. pulchricaudatus* and *P. indicus* each contributed approximately 3%, while *P. hathor* and *P. monodon* made only minor contributions, accounting for 1% and 0.02% of the total number of individuals, respectively. From the 10,859 specimens of *M. monoceros* examined, 6617 were females and 4242 were males. Between the two sampling locations, Jizan accounted for the majority of *M. monoceros* specimens (6098; 56%), while Al Qunfudhah contributed 4761 specimens (44%).

### 3.2. Carapace Length Frequency Distribution

**Figure 2:**
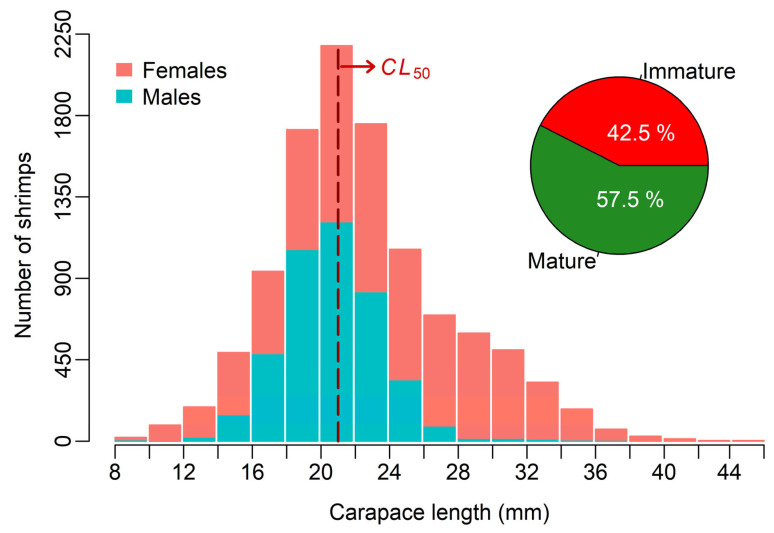
*CL* frequency distribution (in 2 mm intervals) of *M. monoceros* samples from the southeastern Red Sea, stratified by sex. Of 10,859 total sampled specimens, 6617 were females and 4242 were males. *CL*_50_ denotes the *CL* at first sexual maturity for female shrimp, estimated as 21.12 mm. Proportions of immature shrimp (*CL* < *CL*_50_) and mature shrimp (*CL* ≥ *CL*_50_) in the overall catch are also shown.

**Figure 3:**
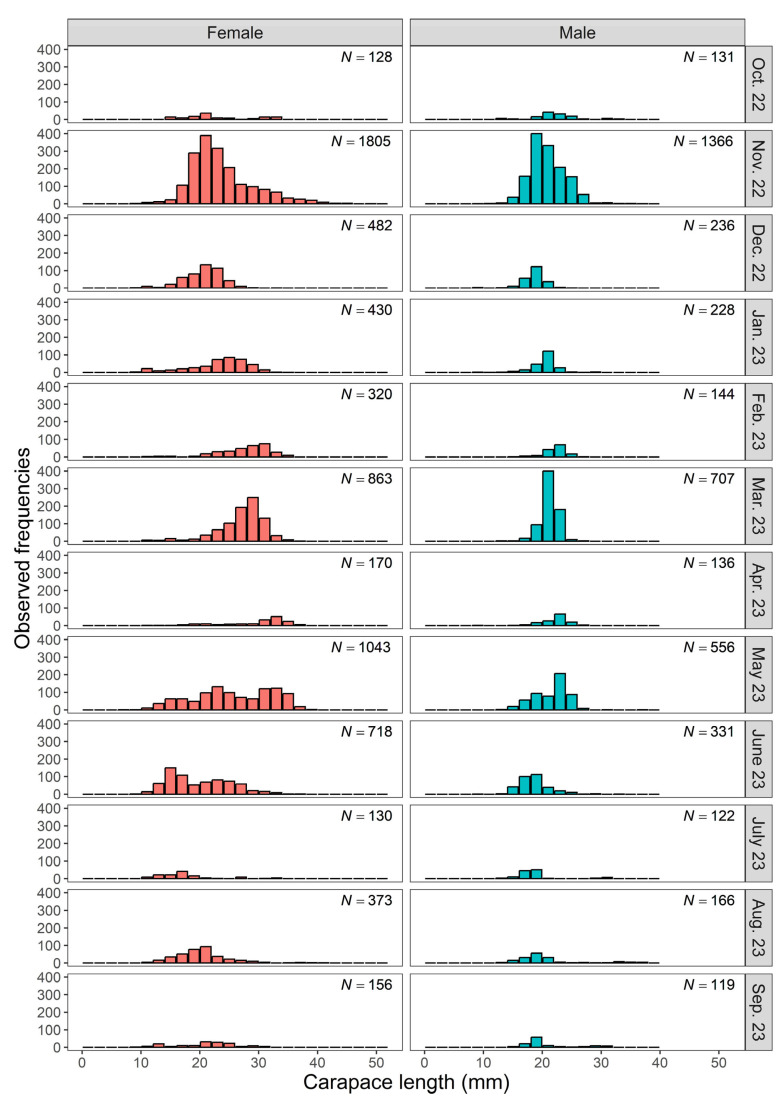
Monthly *CL* frequency distributions of *M. monoceros* by sex.

For all *M. monoceros* samples studied, *CL* ranged from 7.98 to 49.10 mm, while *W* varied between 0.41 and 41.28 g. Summary statistics, including the range, mean, standard deviation (SD), median, and interquartile range (IQR) of *CL* and *W* measurements by sex and combined data, are presented in Table 1. Females exhibited a wider observed range for both *CL* and *W* compared to males (Table 1). Additionally, the variability, as indicated by the SD of both *CL* and *W*, was greater in females, reflecting higher dispersion in these measurements (*F*-test for *CL*, *F* = 3.87, df = 6616, 4241, *p* < 0.001; for *W*, *F* = 5.55, df = 6616, 4241, *p* < 0.001). Females also displayed significantly larger mean values for *CL* and *W* compared to those for males (Table 1, Welch’s approximate *t*-test for *CL*, *t* = 36.26, df = 10,391, *p* < 0.001 and Mann–Whitney test for *W*, *U* = 9,625,587, *p* < 0.001). The overall *CL* frequency distribution of *M. monoceros* samples from the southeastern Red Sea, stratified by sex and grouped into 2 mm class intervals, is illustrated in Figure 2. One female shrimp with a *CL* of 7.98 mm was included in the 8–10 mm class, while another female with a CL of 49.10 mm was grouped into the 44–46 mm class. In addition, the monthly *CL* frequency distributions of *M. monoceros,* separated by sex, are presented in Figure 3. Notable differences in *CL* distributions between males and females were observed across the months, with females generally exhibiting a broader size range and a higher proportion of larger individuals compared to the results for males (Figure 3).

### 3.3. Sex Ratio and Maturity

**Figure 4:**
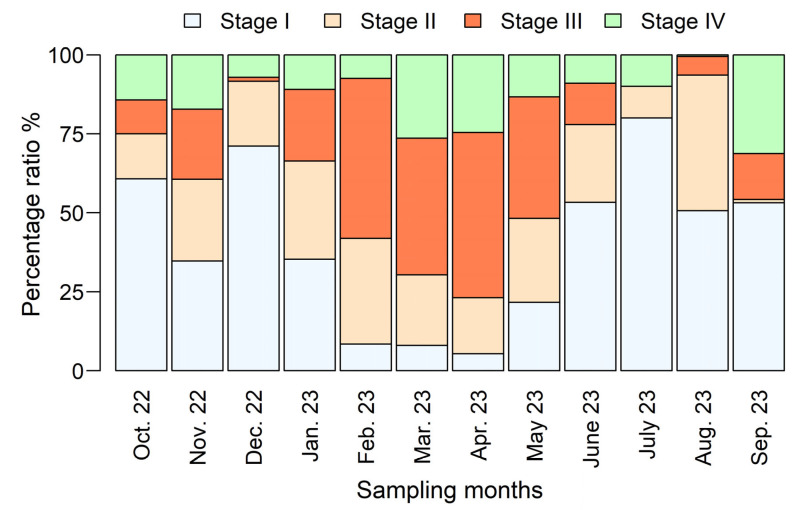
Monthly percentage distribution of female *M. monoceros* maturity stages in the southeastern Red Sea.

The overall sex ratio indicated a predominance of females, at 1.56:1 (Figure 2). The exact binomial test confirmed that this observed sex ratio deviated significantly from unity. The monthly percentage distributions of female speckled shrimp maturity stages are presented in Figure 4. Mature females at Stage III were observed in nearly all sampling months, except for July 2023. They were particularly abundant between February 2023 and May 2023, with the highest proportion (52.3%) was recorded in April 2023 (Figure 4). The smallest mature female at Stage III measured 14.70 mm *CL*, while the mean *CL* of females at this stage was 28.48 ± 4.13 mm.

**Figure 5:**
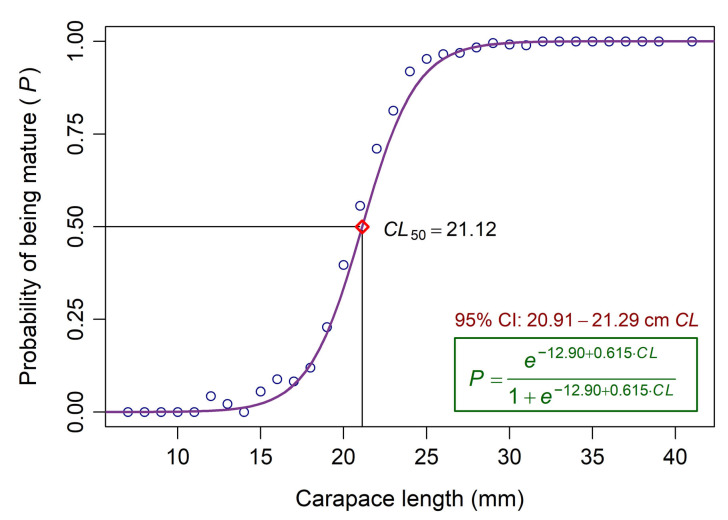
The estimated *CL* at first sexual maturity (*CL_50_*) of female *M. monoceros*. The curve shows the fitted logistic model to the binomial maturity data. Circles represent the observed proportions of mature females relative to the total sampled female shrimp. CI denotes confidence intervals.

The *CL*_50_, estimated by logistic regression analysis, was 21.12 mm for females, with a nonparametric 95% confidence interval of 20.91–21.29 mm (Figure 5). When examining the overall *CL* distribution of the samples, immature speckled shrimp (*CL* < *CL*_50_) accounted for approximately 44% of all specimens caught during the study period (Figure 2). In contrast, mature individuals (*CL* ≥ *CL*_50_) comprised only slightly more than 56% of the total samples.

### 3.4. Length and Weight Relationship

The ANCOVA of *CL* and *W* data revealed no significant differences in the *a* and *b* values between female and male shrimp (ANCOVA, *F* = 0.32, df = 1, 10,855, *p* = 0.58). When all data were combined, the estimated *a* and *b* values (with 95% confidence intervals) for the *CL*–*W* relationship were 0.00236 (0.00229–0.00244) and 2.585 (2.575–2.595), respectively, with a determination coefficient (*r*^2^) of 0.97. The estimated *b* value, significantly lower than 3, indicates a negative allometric growth pattern in this species.

### 3.5. Growth and Mortality

The estimated parameters of the seasonally oscillating von Bertalanffy growth model for females were *CL*_∞_ = 50.66 mm, *K* = 0.35 year^−1^, *t_anchor_* = 0.43 year, *C* = 0.64, and *t_s_* = 0.61 year (Figure S2); for males, they were *CL*_∞_ = 38.97 mm, *K* = 0.55 year^−1^, *t_anchor_* = 0.51 year, *C* = 0.84, and *t_s_* = 0.39 year (Figure S3). The resulting growth performance indices (*φ*′) were 2.96 for females and 2.92 for males. According to the growth parameter estimates, female shrimp grew more slowly than males but reached a larger asymptotic size. Despite the differences in growth characteristics, the overall growth performance remained consistent across sexes.

The *Z* values (with 95% confidence intervals), estimated from the linearized length-converted catch curve analysis, were 2.38 (2.03–2.72) year^−1^ for females and 3.65 (2.66–4.64) year^−1^ for males (Figure S4). The *M* estimates derived from three different methods (in the order previously described) were 1.8, 0.55, and 0.53 year^−1^ for females and 2.7, 0.85, and 0.79 year^−1^ for males. The average *M* values calculated across these methods were 0.96 year^−1^ for females and 1.45 year^−1^ for males. The *F* estimates, based on sex-specific *Z* and *M* values, were 1.42 year^−1^ for females and 2.20 year^−1^ for males.

### 3.6. Stock Evaluation

The *E* values derived from the sex-specific mortality estimates were 0.60 for both female (0.597) and male (0.604) speckled shrimp, exceeding the LRP of 0.5, hence indicating unsustainable fishing pressure.

**Figure 6:**
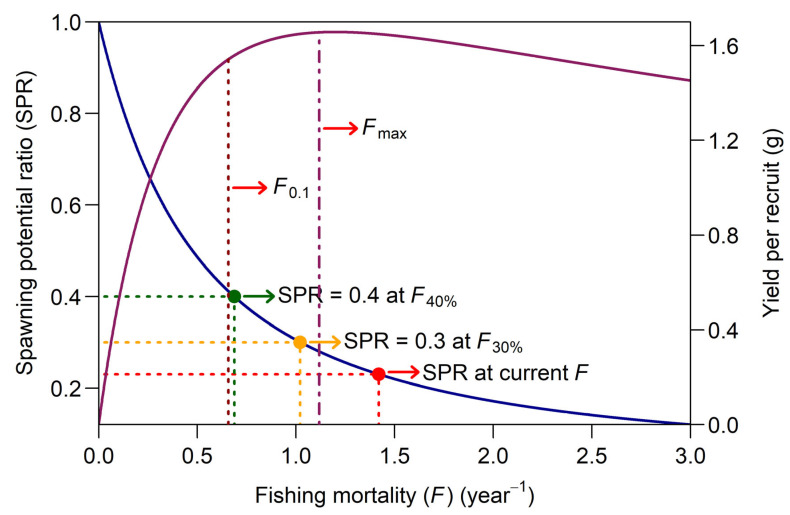
Relationship between SSBpR (dark blue line, corresponding to the left SPR axis) and total YpR (dark red line, corresponding to the right y-axis) as a function of *F*. The SPR and YpR levels were estimated using the Thomson and Bell YpR analysis. The figure also illustrates the SPR values corresponding to the current *F* and various BPRs, including *F_max_*, *F*_0.1_, *F*_40%_, and *F*_30%_.

The BRPs derived from the YpR and SPR analysis for female shrimp are presented in Figure 6. The left y-axis represents SPR, while the right y-axis shows the YpR (g) for a single female shrimp recruiting to the stock. The figure illustrates the decline in SSBpR relative to the unfished level and changes in YpR as a function of *F*. The estimated *F_max_* was 1.20 year^−1^, corresponding to a maximum YpR of 1.66 g. Beyond this *F* level, YpR decreased consistently. The more conservative reference point, *F*_0.1_, was 0.66 year^−1^. The current *F* for female shrimp, estimated at 1.42 year^−1^, substantially exceeded both the *F_max_* and *F*_0.1_. At this *F* value, the SSB was approximately 23% of its unexploited level. The *F*_40%_ value associated with the TRP for SPR analysis was 0.69 year^−1^, and as shown in Figure 6, the *F*_40%_ and *F*_0.1_ estimates were very close.

## 4. Discussion

Marine fishery resources have been declining globally, with developing countries experiencing particularly severe declines due to a combination of overfishing, pollution, increasing market demand, and inadequate management policies. The depletion of these resources poses a significant threat to marine biodiversity, ecosystem stability, and the livelihoods of communities dependent on fisheries. Therefore, it is crucial to implement effective strategies for stock recovery to ensure long-term ecological and economic sustainability. Stock assessment and science-based resource management are essential tools for the conservation and sustainable utilization of living marine resources [67,68,69,70,71]. This study provides new and comprehensive insights into the population dynamics, stock status, and biological characteristics of *M. monoceros* in the southeastern Red Sea. By presenting key population parameters and stock assessment findings, comparisons with similar studies conducted in other regions are facilitated.

The experimental trawl surveys confirmed that *M. monoceros* was the second most abundant shrimp species after *P. semisulcatus*, contributing 10% of the total shrimp catch by number in the southeastern Red Sea. A similar trend was observed by Ghamrawy [30], who reported that this species was also the second most abundant in Jizan, contributing almost 32% of the total shrimp catch. The observed difference in relative abundance between studies may be attributed to temporal variations, including fluctuations in environmental conditions, increased fishing pressure, and changes in ecosystem dynamics over time.

The *CL* frequency distribution for combined sex in the present study ranged from 7.98 mm to 49.1 mm. In comparison, Barua et al. [72] reported a *CL* frequency distribution range of 18–74 mm in Bangladesh. Similarly, Abdallah et al. [28] found that males of *M. monoceros* were predominant within the *TL* range of 70–100 mm, while females became predominant beyond this size range.

The estimated *b* value of 2.58 in the present study indicates a negative allometric growth pattern, meaning that *M. monoceros* grows in *CL* at a faster rate than in *W* for both sexes. This finding aligns with previous studies reporting similar negative allometric growth patterns for this species in different regions. For instance, Sukumaran et al. [13] reported a *b* value of 2.76 along the Indian coast, while Manaşırlı [73] recorded a *b* value of 2.81 in Iskenderun Bay in the northeastern Mediterranean (Turkey). Comparable results were also observed by Lalita Devi [12] and George [18] from India, Ghobashi et al. [74] from the Egyptian Mediterranean, and Mustafa et al. [75] from Bangladesh.

The overall sex ratio in the present study indicated a predominance of females (1.56:1). This pattern is consistent with findings from various regions, where several studies, including those by CMFRI [11], Nandakumar and Srinath [15], Nandakumar [16], Abdallah et al. [17], Abdallah et al. [27,28], Manaşırlı [73], Bayhan et al. [76], and Yılmaz et al. [8] (Table 2), have also reported female-biased sex ratios in *M. monoceros*.

The estimated *CL*_50_ of female speckled shrimp in the present study was 21.12 mm. Ghamrawy [30] reported that *M. monoceros* off the Jizan coast (Saudi Arabia) reached maturity at smaller sizes, with the minimum *CL* to attain Stage III and Stage IV of ovarian maturation being 21 mm and 24 mm, respectively. According to Rao [81], the *TL* at first sexual maturity (*TL*_50_) of *M. monoceros* in the Kakinada area (India) was 96 mm in males and 116 mm in females. Nalini [79] estimated a very close *TL*_50_ value of 118 mm for females off Cochin (southwestern India). George et al. [78] reported that the species reaches maturity at approximately 135 mm *TL* along the Karwar coast (India). Abdel Razek et al. [80] documented the *TL*_50_ for females as 96 mm from the Egyptian Mediterranean coast (Table 2).

Several studies [18,19,20,21,22] have noted that reproduction in *M. monoceros* is characterized by continuous spawning throughout the year, with distinct seasonal peaks. Based on the maturity stages of female gonads observed in the present study (Figure 4), the peak spawning season for this species occurs between February and May in the southern Red Sea. This is further supported by the increased presence of young recruiting individuals, particularly between June and September (Figure 3). Similar findings have been reported in other regions, particularly from the Indian waters. Srivatsa [82] observed that the major spawning season of *M. monoceros* in the Gulf of Kutch occurs from February to April. Sukumaran et al. [13] reported comparable results from the Indian coast, identifying January to April as the peak spawning period. In contrast, Yılmaz et al. [8], who studied the spawning season of speckled shrimp in Antalya Bay (Turkey), reported a different pattern. Contrary to the results of the present study, they found that spawning occurs between December and January. Several other studies have confirmed year-round reproduction in *M. monoceros*. Abdallah et al. [27] and Nalini [79] documented continuous reproduction throughout the year, while Nandakumar [16] reported the presence of advanced mature females year-round along the Kerala coast (India). Additionally, Rao [81] noted that the species follows a bi-monthly spawning cycle, spawning once every two months.

The *CL*_∞_ of female speckled shrimp was estimated at 50.66 mm, while that of males was 38.97 mm in the present study. In contrast, Barua et al. [72] reported a much larger *CL*_∞_ of 85.4 mm for both sexes in Bangladesh. In Karwar, India, Sukumaran et al. [13] estimated the asymptotic *TL* (*TL*_∞_) at 210 mm for females and 180 mm for males, whereas Abdallah et al. [28] reported *TL*_∞_ estimates of 193.3 mm for females and 169.5 mm for males from the Gulf of Gabès in the central Mediterranean Sea (Tunisia). This pattern of sexual dimorphism in size, where females attain a larger asymptotic length than males, has been documented in this species by various researchers across different regions [12,13,23,28,76,78] (Table 2). The *K* estimate in the present study was lower in females than in males, indicating that females reach their *CL*_∞_ at a slower rate compared to males. Despite these differences in growth characteristics, the overall growth performance index remained consistent across sexes.

The *Z* was estimated at 2.38 year^−1^ for females and 3.35 year^−1^ for males in the present study. In comparison, much higher *Z* values have been reported for *M. monoceros* in several other regions, with males frequently exhibiting higher mortality rates than females. For instance, in the Cochin waters of Kerala, the *Z* of *M. monoceros* was estimated at 7.06 year^−1^ for females and 7.75 year^−1^ for males [15]. Similarly, Sukumaran et al. [13] observed higher *Z* values for males across three different regions along both the eastern and western coasts of India, reporting *Z* estimates of 8.05, 5.85, and 4.28 year^−1^ for males, compared to 6.33, 4.68, and 4.17 year^−1^ for females, respectively. Lalita Devi [12], Rao [24], and George et al. [78] also recorded higher *Z* values for male shrimp in Indian waters. In the northeastern Mediterranean Sea, Manaşırlı [73] reported a *Z* value of 5.34 year^−1^ for males, slightly higher than the 5.11 year^−1^ estimated for females. In contrast, findings by Pradana et al. [83] from Indonesia deviated from this pattern, as their study reported a higher *Z* value for females (3.29 year^−1^) than for males (1.9 year^−1^). Overall, the observed sex-based differences in *Z* are likely driven by the combined effects of life history strategies in male penaeid shrimp. Males typically exhibit shorter lifespans than females, a pattern consistent with their faster growth rates and higher natural mortality [36].

The *M* values estimated in the present study were 0.96 year^−1^ for females and 1.45 year^−1^ for males, while the corresponding *F* estimates were 1.42 year^−1^ and 2.20 year^−1^, respectively. Comparatively, Hadj Hamida [77] estimated higher *M* values for *M. monoceros* in the Gulf of Gabès, with 1.73 year^−1^ for females and 2.74 year^−1^ for males. Similarly, Manaşırlı [73] reported higher *M* and *F* values for females in the northeastern Mediterranean Sea, with *M* = 1.65 year^−1^ and *F* = 1.64 year^−1^. Nandakumar and Srinath [15] calculated even higher *F* values, reaching 4.71 year^−1^ for females and 5.40 year^−1^ for males in the Cochin waters.

The current *E*, based on the mortality parameters estimated in the present study, was 0.60 for both sexes, indicating a similar level of fishing pressure on male and female shrimp. This estimate exceeds the LRP of 0.5, clearly signaling a state of overfishing for the *M. monoceros* stock in the southeastern Red Sea. Similar overexploitation trends have been reported for other stocks of this species in different regions. Mwakosya et al. [84] estimated an *E* value of 0.83 in Tanzanian waters, suggesting intense fishing pressure in that region. High *E* values, ranging from 0.60 to 0.85 for males and 0.57 to 0.77 for females, have also been recorded by several authors in Indian waters [12,13,15,78]. In Iskenderun Bay, Manaşırlı [73] reported *E* values of 0.74 for male speckled shrimp and 0.56 for females, further supporting concerns about the overexploitation of this species.

Based on the YpR estimates derived from the Thompson and Bell model analysis for female shrimp (Figure 6), the current *F* of 1.42 year^−1^ substantially exceeds both *F*_0.1_ and *F_max_*, indicating a state of growth overfishing. This suggests that female speckled shrimp are being harvested at a rate that prevents them from reaching their maximum growth potential, thereby reducing YpR and potentially compromising the long-term productivity of the stock. Further evidence of growth overfishing, beyond the YpR analysis, is apparent from the overall *CL* distribution of the sampled shrimp. Nearly half of the catch, a remarkably high proportion, consisted of immature speckled shrimp (*CL* < *CL*_50_) during the study period (Figure 2). If growth overfishing persists, further increases in fishing pressure will likely lead to recruitment overfishing, where the reproductive capacity of the population is impaired. The results of the SPR analysis provide evidence that recruitment overfishing may already be occurring, as the current *F* level reduces the SSB to approximately 23% of its unexploited level, which is below both the TRP and the LRP (Figure 6).

It should be noted, however, that the Thompson and Bell YpR model operates under the assumption of a steady state in the fishery, characterized by a constant fishing pattern over time. This implies that *F* and other input parameters are assumed to have remained relatively stable for a sufficient period, allowing the stock to reach an equilibrium condition. In this study, the absence of a long-term, species-specific time series and reliance on only one year of data present important limitations, particularly for short-lived species like *M. monoceros*. These limitations may introduce biases or uncertainty in the assessment of stock status derived from the YpR model. Therefore, it is essential to continue to expand systematic data collection efforts to enable long-term monitoring and enhance the accuracy of stock assessment.

Even though the YpR and SPR analyses focused solely on females due to their direct contribution to egg production and their critical role in determining the stock’s reproductive potential, the assessment can reasonably be extended to the male component of the population. Males are subject to the same fishing pressure and exploitation level as females, and their removal from the population could indirectly affect reproductive success by altering the operational sex ratio and mating dynamics. An overall evaluation of the current *E* and *F* levels relative to the selected BRPs strongly indicates that the *M. monoceros* stock in the southeastern Red Sea is overexploited. The fishing pressure on both sexes exceeds sustainable levels, posing a significant risk to the long-term viability of the stock and highlighting the urgent need for immediate management interventions. A substantial reduction in *F* to the levels corresponding to either *F*_40%_ or *F*_0.1_ is highly recommended, which would require at least a 50% reduction in fishing effort. This could be achieved by implementing measures such as limiting the number of fishing days, reducing the number of active trawlers, or extending the current five-month seasonal closure for shrimp trawling in both Al Qunfudhah and Jizan to provide additional protection during critical spawning and recruitment periods.

Adjustments to gear selectivity could further contribute to the sustainability of the fishery. Using Bayesian inference, Doll et al. [85] demonstrated that when the length at capture substantially exceeds the length at maturity, both yield and SSB can be maintained at high levels, even under high fishing pressure. The selectivity of the commercial trawl gear presently used in the region needs to be improved, as nearly half of the sampled speckled shrimp in this study consisted of immature individuals (Figure 2). In a recent selectivity study conducted in the Jizan fishing grounds, Santucci et al. [35] showed that square mesh codends were significantly more effective than currently used diamond mesh codends in allowing small, immature *M. monoceros* to escape. Transitioning from diamond to square mesh codends could increase the current *CL_c_* of 17 mm, thereby reducing the proportion of immature individuals in the catch and enhancing the reproductive capacity of the stock.

## 5. Conclusions

This study provides the first comprehensive assessment of the *M. monoceros* fishery in the southeastern Red Sea, encompassing key population parameters and a stock status evaluation. This species was identified as the second most predominant shrimp species, accounting for 10% of the total shrimp catch in the area under investigation. The preliminary stock assessment suggests that current fishing activity in the Saudi Arabian Red Sea exceeds the optimum level and is being conducted at an unsustainable rate, leading to overexploitation. In addition to stock assessment indicators signaling a state of overfishing, nearly half of the total catch of the species consisted of immature individuals, highlighting low gear selectivity as a contributing factor. This underscores the urgent need for immediate management actions to mitigate overfishing and improve sustainability.

Given the economic importance of the shrimp fishery industry, effective management strategies are essential to safeguard its long-term viability. To enhance sustainability, the use of more selective square mesh codends should be implemented to reduce the capture of immature shrimp. Additional measures, such as limiting the number of fishing days, reducing the number of active trawlers, introducing catch quotas, as well as extending the current five-month seasonal closure for trawling, should be considered to provide further protection during critical spawning and recruitment periods.

It is imperative to maintain robust data collection efforts for continuous monitoring and assessment. Additionally, integrating environmental variables into the modeling framework will be essential for a comprehensive understanding of population dynamics. Given the potential impacts of climate change on marine living resources, future research should incorporate climate-related factors into management strategies to ensure the long-term sustainability of the *M. monoceros* fishery.

All these recommended measures and monitoring efforts should be embedded within a multiannual, ecosystem-based management plan developed in collaboration with relevant stakeholders. Such a plan would provide a coordinated framework for implementing regulatory actions, enhancing data collection, and supporting adaptive management through continuous stock assessment and environmental monitoring.

## Figures and Tables

**Table 1:** Descriptive statistics for carapace length (*CL*) and weight (*W*) of *M. monoceros* collected from the southeastern Red Sea, presented for the total sample and separated by sex. *N*, SD, and IQR denote sample size, standard deviation, and interquartile range, respectively.

Sex	*N*	Carapace Length (mm)				Weight (g)			
		Range	Mean ± SD	Median	IQR	Range	Mean ± SD	Median	IQR
Female	6617	7.98–49.10	23.76 ± 5.83	23.32	19.70–28.00	0.41–41.28	9.52 ± 5.71	8.35	5.04–13.44
Male	4242	8.40–37.56	20.68 ± 2.96	20.60	18.70–22.42	0.46–25.88	6.21 ± 2.42	6.08	4.60–7.50
Total	10,859	7.98–49.10	22.56 ± 5.14	21.75	19.22–25.30	0.41–41.28	8.29 ± 5.02	7.00	4.78–10.52

**Table 2:** Summary of maximum size, sex ratio, size at first maturity, and von Bertalanffy growth parameters estimated for *M. monoceros* in different regions of the world. M and F denote males and females, respectively. When a value is reported for combined sexes, it is presented in between the M and F columns. Measurements based on *CL* rather than *TL* are marked with an asterisk (*).

Location	Max Size (mm)		Sex Ratio (F:M)	*TL*_50_ (mm)		*TL*_∞_ (mm)		*K* (Year^−1^)		*W*_∞_ (g)		References
	M	F		M	F	M	F	M	F	M	F	
Tunisia			1.5:1	76.5	122.3							Abdallah et al. [27]
			1.7:1			169.5	193.3	2.06	1.36			Abdallah et al. [28]
	160	178		76.7	117.6							Hadj Hamida [77]
India	199	226	1.25:1	105.4	121.3	208.9	237.3	1.6	1.5			CMFRI [11]
						208.4	216.2	1.0	1.0			Lalitha Devi [12]
				96	115.5	180	210	1.8	1.8	37	71	Sukumaran et al. [13]
	163	187				178.4	207.3	1.68	1.62	36	68	Rao and Krishnamoorthi [23]
				135.5		190	225					George et al. [78]
				118								Nalini [79]
Egypt				96								Abdel Razek et al. [80]
Turkey	145	165	2.8:1	75	115							Yılmaz et al. [8]
	150	155	1.4:1			162.8	178.4	1.39	1.51			Manaşırlı [73]
	155	190	1.3:1			157.5	199.5	0.71	0.52	26	40	Bayhan et al. [76]
Bangladesh	74 *			33.3 *		85.4 *						Barua et al. [72]
Red Sea, Saudi Arabia	37.6 *	49.1 *	1.56:1		21.12 *	38.97 *	50.66 *	0.55	0.35	31	60	Present study

## Data Availability

The data presented in this study are available on request from the corresponding author (The data are not publicly available due to privacy restrictions set by the funding institution).

## Supplementary Materials

The following supporting information can be downloaded at: https://www.mdpi.com/article/10.3390/biology14040406/s1, Figure S1: Ovarian maturation stages in *M. monoceros*: (a) Stage I—Immature or undeveloped; (b,c) Stage II—Developing; (d,e) Stage III—Mature; (f) Stage IV—Spent; Figure S2: (a) Monthly carapace length frequency distributions of female *M. monoceros* from the southeastern Red Sea, using a bin size of 2 mm; (b) restructured length frequency data with a moving average applied over five carapace length classes (bins), along with the seasonally oscillating von Bertalanffy growth curves (dashed dark red curves). Both plots were generated using the TropFishR package in R; Figure S3: (a) Monthly carapace length frequency distributions of male *M. monoceros* from the southeastern Red Sea, using a bin size of 2 mm; (b) restructured length frequency data with a moving average applied over five carapace length classes (bins), along with the seasonally oscillating von Bertalanffy growth curves (dashed dark red curves). Both plots were generated using the TropFishR package in R; Figure S4: Linearized length-converted catch curve analysis for estimating the total mortality rate (Z) of *M. monoceros*, based on simple linear regression analysis: (a) females and (b) males. CI denotes the confidence interval of *Z*. The shaded grey area around each regression line represents the 95% confidence bands.

## References

[1] Crosnier A. (1965). Les Crevettes Penaeides du Plateau Continental Malgache. Cah. O.R.S.T.O.M. Ser. Océanogr..

[2] Racek A.A., Dall W. (1965). Littoral *Penaeinae* (*Crustacea Decapoda*) from Northern Australia, New Guinea and adjacent waters. Verh. K. Ned. Akad. Wet. II.

[3] Fischer W., Bianchi G. (1984). FAO Species Identification Sheets for Fishery Purposes. Western Indian Ocean (Fishing Area 51). Prepared and Printed with the Support of the Danish International Development Agency (DANIDA).

[4] Balss H. (1927). Bericht über die Crustacea Decapoda (Natantia und Anomura). Zoological results of the Cambridge Expedition to the Suez Canal, 1924. XIV. Trans. Zool. Soc. London.

[5] Holthuis L.B., Fisher W., Bauchot M.L., Schneider M. (1987). Invertébrés Marins: *Les Crevettes*. Fiches FAO D’identification des Espèces Pour les Besoins de la Pêche. Zone 37. Révision.

[6] Galil B., Froglia C., Nöel P., Briand F. (2002). CIESM Atlas of Exotic Species in the Mediterranean. Crustaceans: Decapods and Stomatopods.

[7] Can M.F., Mazlum Y., Demirci A., Aktaş M. (2004). The catch composition and catch per unit of swept area (CPUE) of penaeid shrimps in the bottom trawls from Iskenderun Bay, Turkey. Turk. J. Fish. Aquat. Sci..

[8] Yılmaz S., Özvarol Z.A.B., Özvarol Y. (2009). Fisheries and Shrimp Economy, Some Biological Properties of the Shrimp *Metapenaeus monoceros* (Fabricius, 1798) in the Gulf of Antalya (Turkey). J. Anim. Vet. Adv..

[9] FAO (2001). Tropical Shrimp Fisheries and their Impact on Living Resources. Shrimp Fisheries in Asia: Bangladesh, Indonesia and the Philippines; in the Near East: Bahrain and Iran; in Africa: Cameroon, Nigeria and the United Republic of Tanzania; in Latin America: Colombia, Costa Rica, Cuba, Trinidad and Tobago, and Venezuela, Fishery Technology Service, Fishery Industries Division. https://www.fao.org/4/y2859e/y2859e01.htm.

[10] Holthuis L.B. (1980). FAO Species Catalogue. Vol. 1. Shrimps and prawns of the world. An annotated catalogue of species of interest to fisheries. Crustacea FAO Fish. Synop..

[11] CMFRI (2017). Annual Report 2016-17.

[12] Lalita Devi S. (1987). Growth and Population Dynamics of the three Penaeid Prawns in the Trawling Grounds off Kakinada. Indian J. Fish..

[13] Sukumaran K.K., Deshmukh V.D., Rao G.S., Alagaraja K., Sathianandan T.V. (1993). Stock assessment of the penaeid prawn *Metapenaeus monoceros* Fabricius along the Indian Coast. Indian J. Fish..

[14] Rao G.S. (1988). Studies on the feeding biology of *Metapenaeus monoceros* (Fabricius) along the Kakinada coast. J. Mar. Biol. Ass. India.

[15] Nandakumar G., Srinath M. (1999). Stock assessment of *Metapenaeus monoceros* (Fabricius) from Cochin Waters, Kerala. Indian J. Fish..

[16] Nandakumar G. (2001). Reproductive biology of the speckled shrimp *Metapenaeus monoceros* (Fabricius). Indian J. Fish..

[17] Abdallah O.B.H.H.B., Jarboui O., Missaoui H., Hadj Hamida N.B. (2003). Croissance relative, sex-ratio et exploitation de la crevette blanche *Metapenaeus monoceros* (Fabricius, 1798) du golfe de Gabès (Tunisie). Bull. De L’institut Natl. Sci. Technol. Mer Salammbô.

[18] George M.J. (1962). On the breeding of penaeids and the recruitment of their post-larvae into the backwaters of Cochin. Indian J. Fish..

[19] Gopalakrishnan V. (1971). The biology of the Hooghly Matalah estuarine system (West Bengal, India) with special reference to its fisheries. J. Mar. Biol. Ass. India.

[20] Subrahmanyam M., Ganapati P.N. (1971). Observations on the post-larval prawns from the Godavari estuarine systems (Andhra Pradesh, India) with notes on their role in capture and culture fisheries. J. Mar. Biol. Ass. India.

[21] Thomas M.M. (1974). Reproduction, fecundity and sex ratio of the green tiger prawn, *Penaeus semisulcatus* de Haan. Indian J. Fish..

[22] Ghamrawy M.S. (1982). Studies on the Ecology and Biology of Penaeid Shrimp in the Region of Jeddah, Saudi Arabia. Ph.D. Thesis.

[23] Rao G.S., Krishnamoorthi B. (1990). Age and growth of *Metapenaeus monoceros* along the Kakinada Coast. J. Mar. Biol. Ass. India.

[24] Rao G.S. (1994). Mortality rates and stock assessment of *Metapenaeus monoceros* along the Kakinada coast. J. Mar. Biol. Ass. India.

[25] Dineshbabu A.P., Zacharia P.U., Thomas S., Kizhakudan S.J., Rajesh K.M., Vivekanandan E., Pillai S.L., Sivadas M., Ghosh S., Ganga U. (2020). Assessment of stock vulnerability of Indian marine fishes to past changes in climate and options for adaptation. Clim. Res..

[26] Missaoui H., Zaouali J. (1995). Apparition de Nouveaux Crustacés Dans les pêches Crevettières du Golfe de Gabès, Tunisie. Mar. Life.

[27] Abdallah O.B.H.H.B., Hamida N.B.H., Jarboui O., Fiorentino F., Missaoui H. (2009). Reproductive biology of the speckled shrimp *Metapenaeus monoceros* (Fabricius, 1798) (Decapoda: Penaeidae) in the Gulf of Gabès (Southern Tunisia, Eastern Mediterranean). Cah. Biol. Mar..

[28] Abdallah O.B.H.H.B., Hamida N.B.H., Jarboui O., Missaoui H. (2010). Age and growth of the speckled shrimp *Metapenaeus monoceros* (Fabricius, 1798) in the Gulf of Gabès (Southern Tunisia, Central Mediterranean). Cah. Biol. Mar..

[29] Alam M.S., Liu Q., Schneider P., Mozumder M.M.H., Uddin M.M., Monwar M.M., Hoque M.E., Barua S. (2022). Stock Assessment and Rebuilding of Two Major Shrimp Fisheries (*Penaeus monodon* and *Metapenaeus monoceros*) from the Industrial Fishing Zone of Bangladesh. J. Mar. Sci. Eng..

[30] Ghamrawy M.S. (1990). Notes on the Biology of Penaeid Shrimp at Gizan, Red Sea. J.K.A.U. Mar. Sci..

[31] MEWA (2023). The Statistical Book. https://www.mewa.gov.sa/ar/InformationCenter/Researchs/Reports/GeneralReports/%d8%a7%d9%84%d9%83%d8%aa%d8%a7%d8%a8%20%d8%a7%d9%84%d8%a5%d8%ad%d8%b5%d8%a7%d8%a6%d9%8a%202023%d9%85.pdf.

[32] Kadengal S.T., Ceyhan T., Tosunoğlu Z., Gireesh S., Charles S.K., Santucci R.G., Adam A.M.S., Tıraşın E.M., Ünal V., Dimech M. (2024). Toward Sustainable Fisheries: Assessing Catch per Unit Effort, Retained Bycatch, and Discard Ratios in the Red Sea Shrimp Trawl Fishery of the Kingdom of Saudi Arabia. Sustainability.

[33] MEWA Implementing Regulation of the Agriculture Law Issued by Royal Decree No (M/64) Dated 10/8/1442 Hijri. 23 March 2021. https://www.mewa.gov.sa/en/InformationCenter/DocsCenter/RulesLibrary/Docs/Implementing%20Regulations%20of%20The%20Agriculture%20Law.pdf.

[34] KAUST Beacon Development (KBD) (2023). Assessment and Management of Shrimp Trawl Fisheries in the Red Sea Waters of the Kingdom. Report for the Ministry of Environment, Water and Agriculture (MEWA).

[35] Santucci R.G., Tosunoğlu Z., Cilbiz M., Charles S.K., Gireesh S., Kadengal S.T., Adam A.M.S., Tıraşın E.M., Ünal V., Dimech M. (2024). Assessing Codend Mesh Selectivity: Comparing Diamond and Square Mesh Codend in the Red Sea Shrimp Trawl Fishery of Saudi Arabia. J. Mar. Sci. Eng..

[36] Bauer R.T. (2023). Shrimps: Their Diversity, Intriguing Adaptations and Varied Lifestyles.

[37] Amanat Z., Qureshi N.A. (2011). Ovarian Maturation Stages and Size at Sexual Maturity of *Penaeus indicus* (H. Milne Edwards, 1937) in the Lagoon Water of Sonmiani Bay, Balochistan. Pakistan J. Zool..

[38] Sokal R.R., Rohlf F.J. (2012). Biometry. The Principles and Practice of Statistics in Biological Research.

[39] Quinn T.J., Deriso R.B. (1999). Quantitative Fish Dynamics.

[40] Tıraşın E.M. (1993). Balık populasyonlarının büyüme parametrelerinin araştırılması. Turkish J. Zool..

[41] Hosmer D.W., Lemeshow S., Sturdivant R.X. (2013). Applied Logistic Regression.

[42] Aydın C.M., Tıraşın E.M. (2023). Information on the deep-water giant red shrimp *Aristaeomorpha foliacea* (Risso, 1827) (Crustacea, Decapoda, Aristeidae) population in Antalya Bay (Eastern Mediterranean Sea, South of Turkey) based on the MEDITS protocol. Reg. Stud. Mar. Sci..

[43] Efron B., Tibshirani R.J. (1993). An Introduction to the Bootstrap.

[44] Somers I.F. (1988). On a seasonally oscillating growth function. Fishbyte.

[45] Pauly D., Pauly D., Morgan G.R. (1987). A Review of the ELEFAN System for Analysis of Length-Frequency Data in Fish and Aquatic Invertebrates. Length-Based Methods in Fisheries Research.

[46] Beverton R.J.H., Holt S.J. (1957). On the Dynamics of Exploited Fish Populations.

[47] Mildenberger T.K., Taylor M.H., Wolff M. (2017). TropFishR: An R package for fisheries analysis with length-frequency data. Methods Ecol. Evol..

[48] Taylor M.H., Mildenberger T.K. (2017). Extending electronic length frequency analysis in R. Fish. Manag. Ecol..

[49] Pauly D., David N. (1981). ELEFAN I, a BASIC Program for the Objective Extraction of Growth Parameters from Length-Frequency Data. Meeresforsch.

[50] Pauly D., Munro J.L. (1984). Once More on the Comparison of Growth in Fish and Invertebrates. Fishbyte.

[51] Sparre P., Venema S.C. (1998). Introduction to Tropical Fish Stock Assessment. FAO Fisheries Technical Papers, No. 306/1, Rev. 2 (Part 1).

[52] Cope J.M., Hamel O.S. (2022). Upgrading from M version 0.2: An application-based method for practical estimation, evaluation and uncertainty characterization of natural mortality. Fish. Res..

[53] Maunder M.N., Hamel O.S., Lee H.H., Piner K.R., Cope J.M., Punt A.E., Ianelli J.N., Castillo-Jordán C., Kapur M.S., Methot R.D. (2023). A review of estimation methods for natural mortality and their performance in the context of fishery stock assessment. Fish. Res..

[54] Hamel O.S., Cope J.M. (2022). Development and considerations for application of a longevity-based prior for the natural mortality rate. Fish. Res..

[55] Then A.Y., Hoenig J.M., Hall N.G., Hewitt D.A. (2015). Evaluating the predictive performance of empirical estimators of natural mortality rate using information on over 200 fish species. ICES J. Mar. Sci..

[56] Goodyear C.P. (1993). Spawning stock biomass per recruit in fisheries management: Foundation and current use. Can. Spec. Publ. Fish. Aquat. Sci..

[57] Gabriel W.L., Sissenwine M.P., Overholtz W.J. (1989). Analysis of spawning stock biomass per recruit: An example for Georges Bank haddock. N. Am. J. Fish. Manag..

[58] Caddy J.F., Mahon R. (1995). Reference Points for Fisheries Management; FAO Fisheries Technical Paper 347.

[59] Caddy J.F., McGarvey R. (1996). Targets or limits for management of fisheries?. N. Am. J. Fish. Manag..

[60] Prager M.H., Porch C.E., Shertzer K.W., Caddy J.F. (2003). Targets and limits for management of fisheries: A simple probability-based approach. N. Am. J. Fish. Manag..

[61] Clark W.G. (2002). *F*_35%_ revisited ten years later. N. Am. J. Fish. Manag..

[62] Mace P.M. (1994). Relationships between common biological reference points used as thresholds and targets of fisheries management strategies. Can. J. Fish. Aquat. Sci..

[63] Mace P.M., Sissenwine M.P. (1993). How Much Spawning per Recruit is Enough?. Canadian Special Publication of Fisheries and Aquatic Sciences.

[64] Walters C., Martell S.J.D. (2004). Fisheries Ecology and Management.

[65] Froese R., Winker H., Gascuel D., Sumaila U.R., Pauly D. (2016). Minimizing the impact of fishing. Fish Fish..

[66] R Core Team (2024). R: A Language and Environment for Statistical Computing. R Foundation for Statistical Computing.

[67] Butterworth D.S., Johnston S.J., Brandao A. (2010). Pretesting the Likely Efficacy of Suggested Management Approaches to Data-Poor Fisheries. Mar. Coast. Fish..

[68] Watson R.A., Cheung W.W.L., Anticamara J.A., Sumaila R.U., Zeller D., Pauly D. (2013). Global marine yield halved as fishing intensity redoubles. Fish Fish..

[69] Pauly D., Zeller D. (2016). Catch reconstructions reveal that global marine fisheries catch are higher than reported and declining. Nat. Commun..

[70] Benson A.J., Stephenson R.L. (2018). Options for integrating ecological, economic, and social objectives in evaluation and management of fisheries. Fish Fish..

[71] Punt A.E. (2019). Spatial stock assessment methods: A viewpoint on current issues and assumptions. Fish. Res..

[72] Barua S., Liu Q., Alam M.S., Schneider P., Chowdhury S.K., Mozumder M.M.H. (2023). Assessment of Three Major Shrimp Stocks in Bangladesh Marine Waters Using Both Length-Based and Catch-Based Approaches. Sustainability.

[73] Manaşırlı M. (2014). Population Dynamics of the *Metapenaeus monoceros* (Fabricius, 1798) in North-eastern Mediterranean Sea. Iran. J. Fish. Sci..

[74] Ghobashi A.F.A., Abdel Razek F.A., Khafage A.R., Taha S.M. (2009). Biological studies of Red shrimp *Metapenaeus monoceros* (Fabricius) in Egyptian Mediterranean waters 1- Morphometric characteristics. Egypt. J. Aquat. Res..

[75] Mustafa M.G., Ali M.S., Azadi M.A. (2006). Some Aspect of Population Dynamics of Three Penaeid Shrimps (*Penaeus monodon*, *Penaeus semisulcatus* and *Metapenaeus monoceros*) from the Bay of Bengal, Bangladesh. Chittagong Univ. J. Sci..

[76] Bayhan Y.K., Ünlüer T., Özdöl M., Ataoğlu H., Çiçek E. (2023). Growth, reproduction, and mortality rates of *Metapenaeus monoceros* (Fabricius, 1798) in 1981-1982 from the north-eastern Mediterranean coasts of Turkey. Cah. Biol. Mar..

[77] Hadj Hamida O.B.A.B. (2012). Etude biologique et dynamique de la crevette mouchetée *Metapenaeus monoceros* (Fabricius, 1798) exploitée dans le golfe de Gabès, Tunisia. Ph.D. Thesis.

[78] George M.J., Alagaraja K., Sukumaran K.K., Nandakumar G., Ramamurthy S., Telang K.Y. (1988). The present status of shrimp trawling and its impact on shrimp stocks of Karnataka Coast. Seminar Proceedings Problems and Prospects of Marine Fishing and Fish Processing in Karnataka.

[79] Nalini C. (1976). Observations on the Maturity and Spawning of *Metapenaeus monoceros* (Fabricius) at Cochin. Indian J. Fish..

[80] Abdel Razek F.A., Ghobashi A.F.A., Khafage A.R., Taha S.M. (2009). Biological studies of Red shrimp *Metapenaeus monoceros* (Fabricius) in Egyptian Mediterranean waters 2-Maturation and Spawning. Egypt. J. Aquat. Res..

[81] Rao G.S. (1989). Studies on the reproductive biology of the brown prawn *Metapenaeus monoceros* (Fabricius: 1798) along the Kakinada coast. Indian J. Fish..

[82] Srivatsa K.R. (1953). A Survey and Comparative Analysis of the Prawn (Shrimp) Fishery of the Gulf of Kutch in Saurashtra, India.

[83] Pradana R.T., Syahrir R.M., Abdunnur A. (2023). Population Dynamics of Yellow Prawn (*Metapenaeus monoceros*) Captured at Night in the Waters of Samboja Kuala, Kutai Kartanegara Regency. Nusant. Trop. Fish. Sci. (Ilmu Perikan. Trop. Nusant.).

[84] Mwakosya C.A., Kuguru B., Senkondo E., Kulekana J.J., Ulotu E., Sululu J., Mhitu H., Matola H., Budeba Y.L., Ngatunga B.P. (2010). The Assessment of the Effectiveness of Closure of Commercial Prawn Trawling in Tanzania Coastal Waters. Report for MACEMP Project Submitted to Ministry of Livestock and Fisheries Development. https://www.researchgate.net/publication/265133904.

[85] Doll J.C., Lauer T.E., Clark-Kolaks S. (2017). Yield-per-recruit modeling of two piscivores in a Midwestern reservoir: A Bayesian approach. Fish. Res..

