# Supplementary material for: Life History Traits and Fishery Dynamics of Speckled Shrimp, Metapenaeus monoceros (Fabricius, 1798), Along the Saudi Arabian Red Sea Coast
Source: Biology (Basel). 2025 Apr 11;14(4):406. doi: 10.3390/biology14040406 (PMC12024536; doi:10.3390/biology14040406)
Supplement: Supplementary file 1 [file biology-14-00406-s001.zip › biology-3545373-supplementary.pdf]

biology

Supplementary materials

MDPI

Article

Life History Traits and Fishery Dynamics of Speckled Shrimp, *Metapenaeus monoceros* (Fabricius, 1798) along the Saudi Arabian Red Sea Coast

Sheeja Gireesh [1], Eyüp Mümtaz Tıraşın [1,2]*, Goutham Bharathi Muthu Palani [1], Santhosh Kumar Charles [1], Sirajudheen Thayyil Kadengal [1], Ronald Grech Santucci [1], Ricardo Clapis Garla [1], Zahra Okba [1], Adel M. S. Adam [1] and Mark Dimech [1]

[1] KAUST Beacon Development Department, National Transformation Institute, King Abdullah University of Science and Technology, Thuwal 23955-6900, Saudi Arabia; sheeja.gireesh@kaust.edu.sa (S.G.), goutham.muthupalani@kaust.edu.sa (G.B.M.P.), santhosh.charles@kaust.edu.sa (S.K.C.), sirajudheen.kadengal@kaust.edu.sa (S.T.K.), ronald.grechsantucci@kaust.edu.sa (R.G.S.), ricardo.garla@kaust.edu.sa (R.C.G.), zahra.okba@kaust.edu.sa (Z.O.), adel.adam@kaust.edu.sa (A.M.S.A.), mark.dimech@kaust.edu.sa (M.D.)
[2] Institute of Marine Sciences and Technology, Dokuz Eylül University, İnciraltı 35340, İzmir, Türkiye
* Correspondence: eyupmumtaz.tirasin@kaust.edu.sa

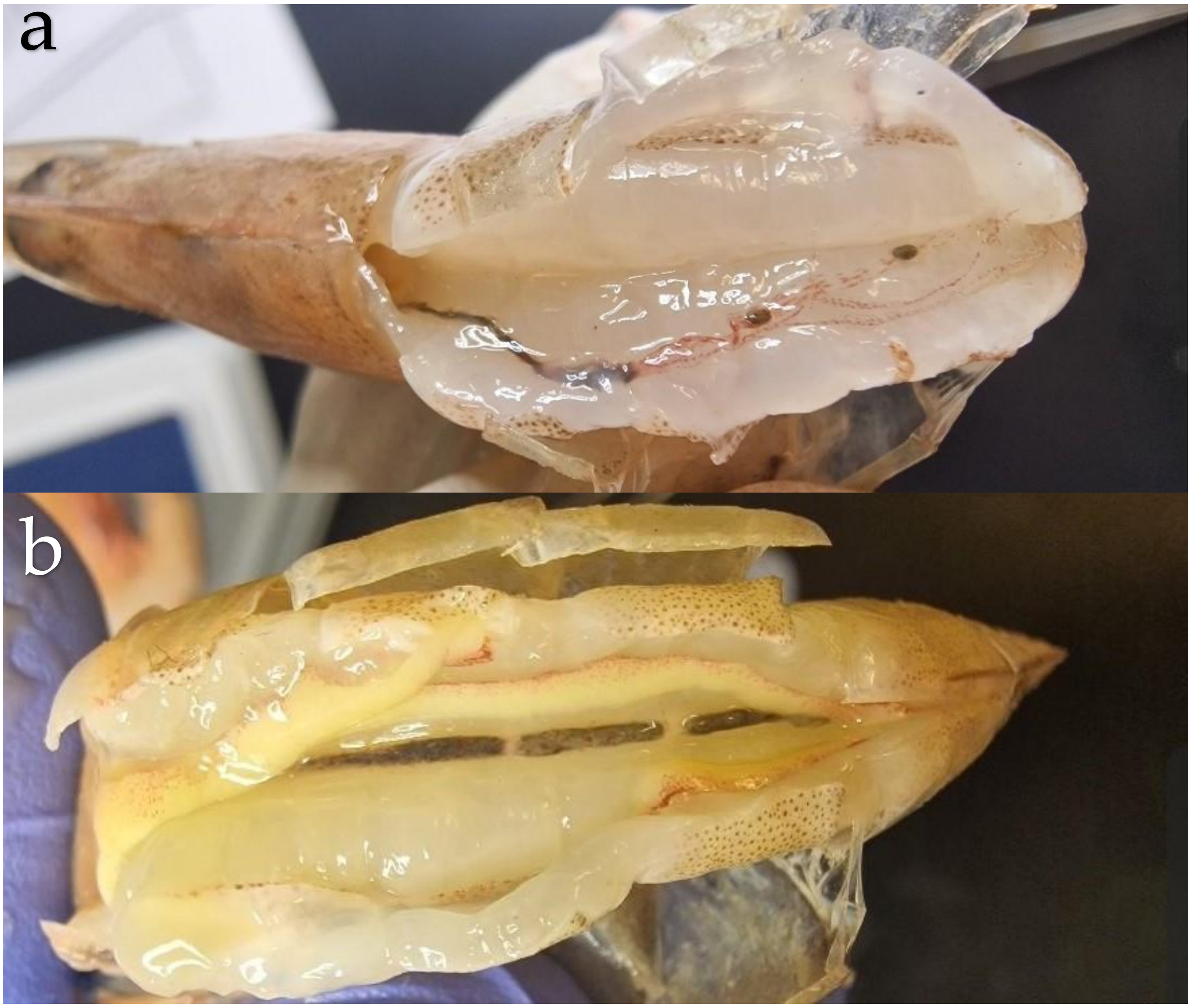

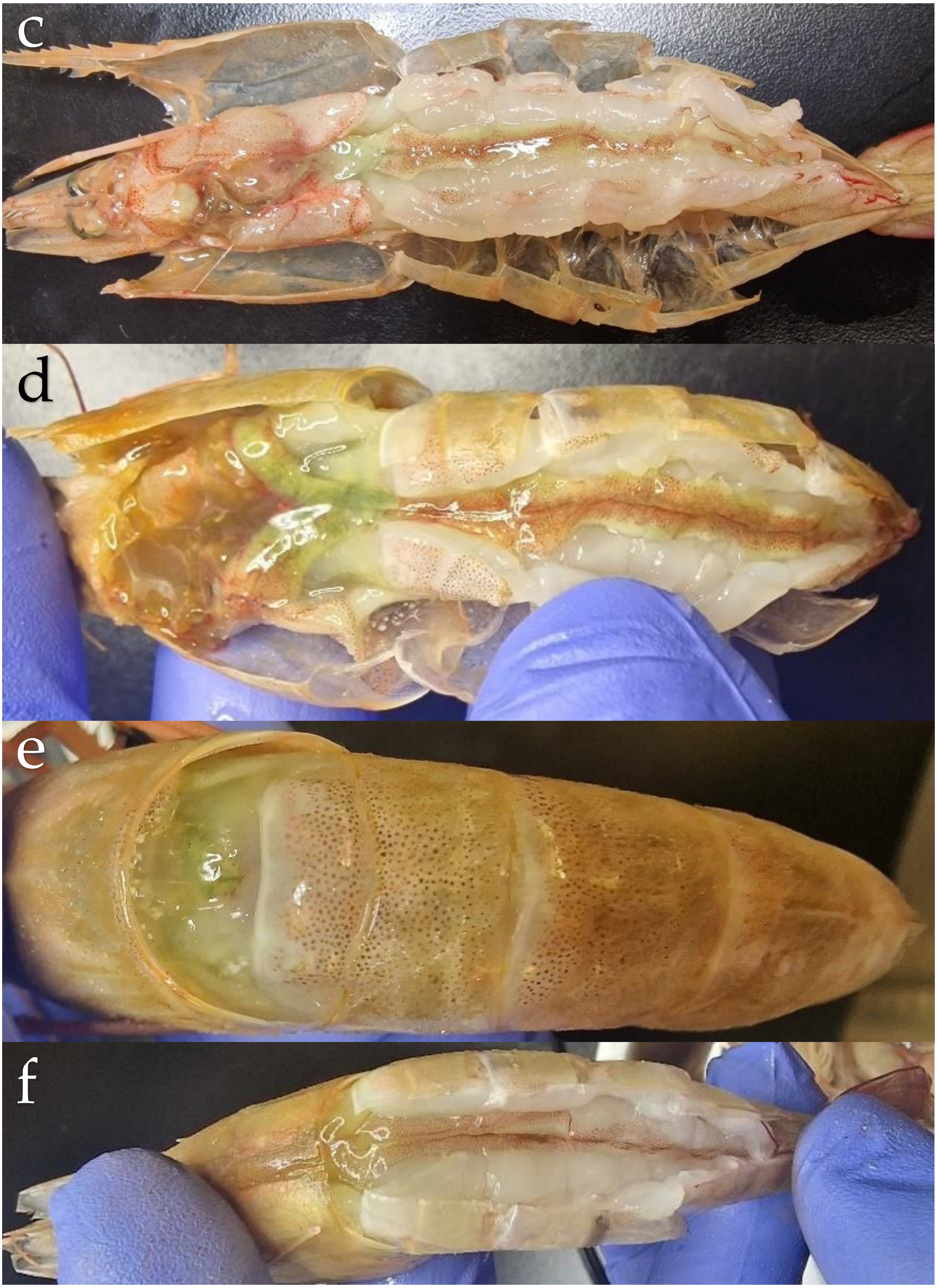

Figure S1. Ovarian maturation stages in *M. monoceros*: (a) Stage I – Immature or undeveloped; (b, c) Stage II – Developing; (d, e) Stage III – Mature; (f) Stage IV – Spent.

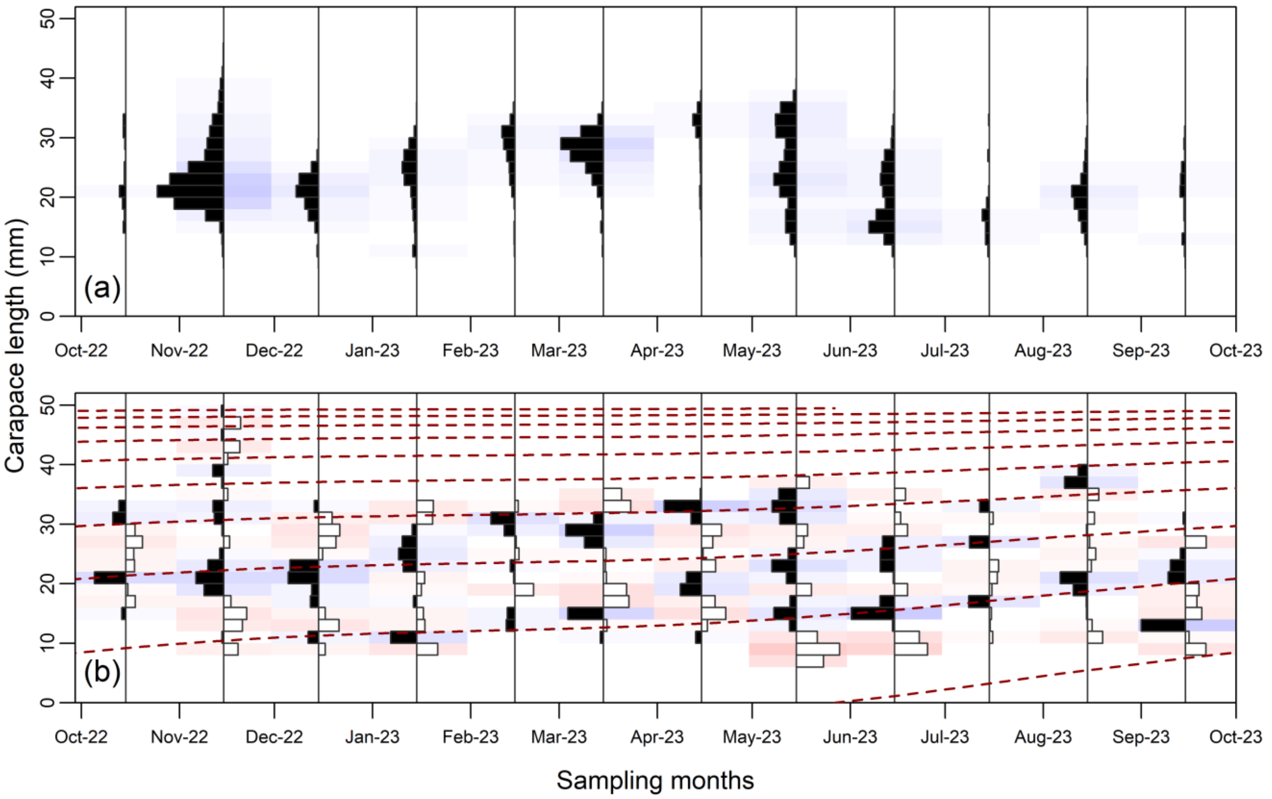

Figure S2. (a) Monthly carapace length frequency distributions of female *M. monoceros* from the southeastern Red Sea, using a bin size of 2 mm; (b) restructured length frequency data with a moving average applied over five carapace length classes (bins), along with the seasonally oscillating von Bertalanffy Growth Curves (dashed dark red curves). Both plots were generated using the TropFishR package in R.

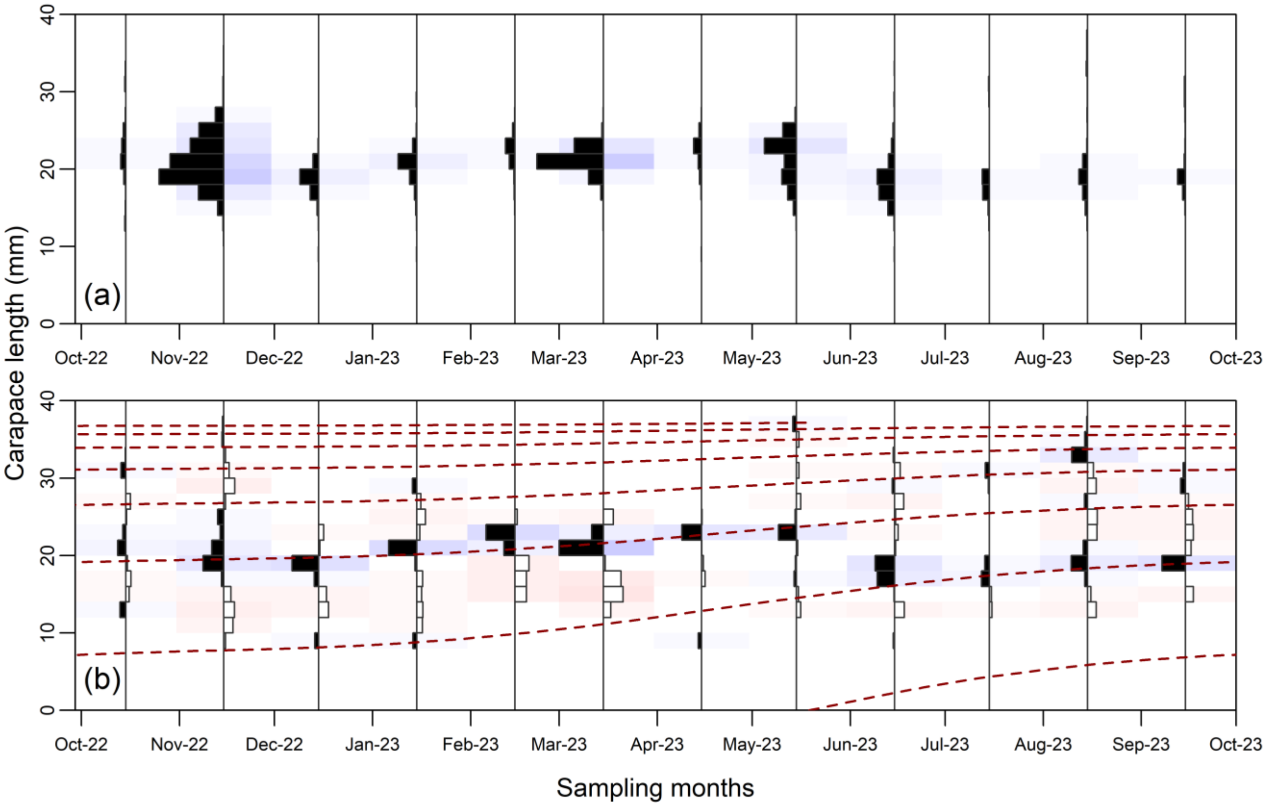

Figure S3. (a) Monthly carapace length frequency distributions of male *M. monoceros* from the southeastern Red Sea, using a bin size of 2 mm; (b) restructured length frequency data with a moving average applied over five carapace length classes (bins), along with the seasonally oscillating von Bertalanffy Growth Curves (dashed dark red curves). Both plots were generated using the TropFishR package in R.

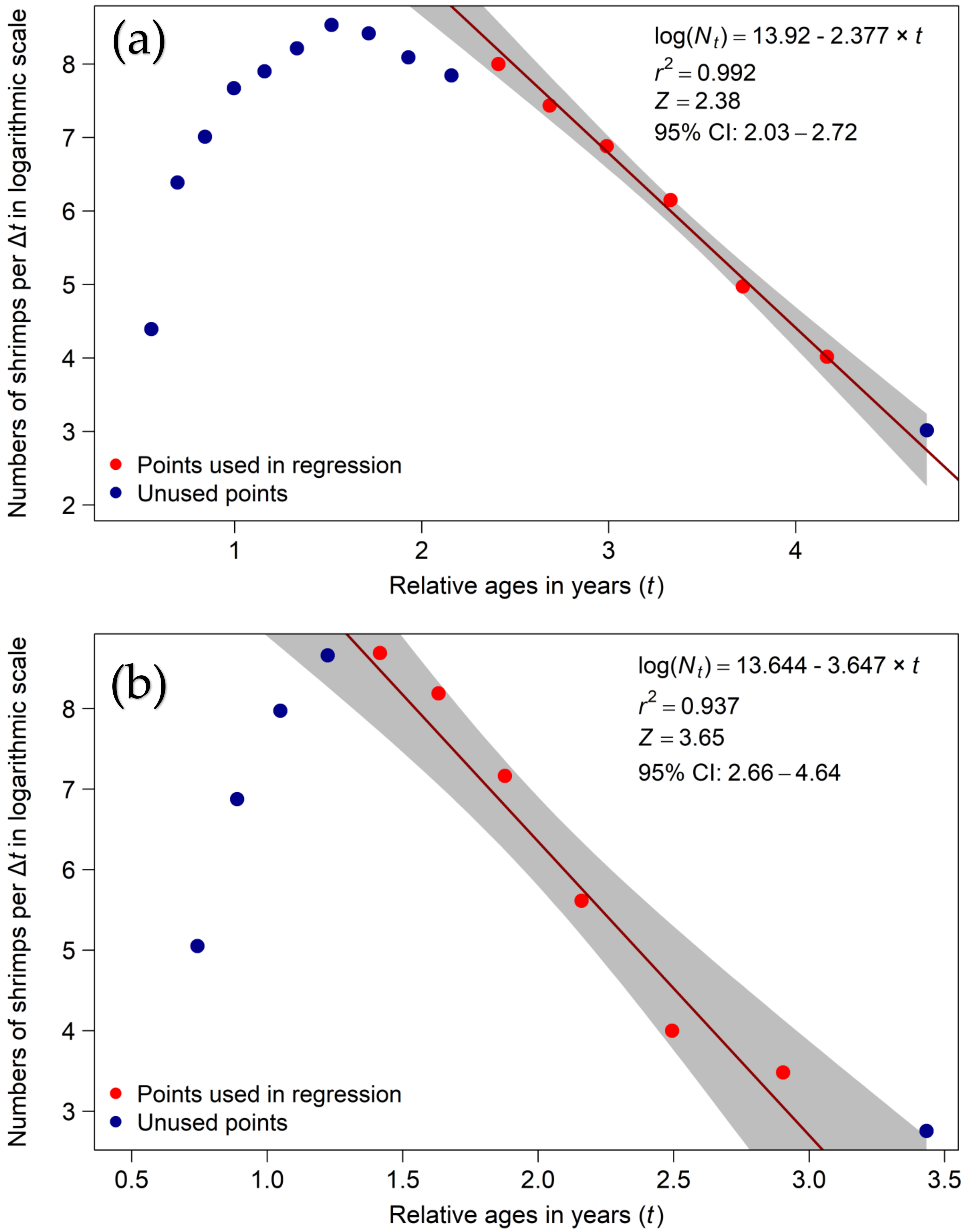

Figure S4. Linearized length-converted catch curve analysis for estimating the total mortality rate (Z) of *M. monoceros*, based on simple linear regression analysis: (a) females and (b) males. CI denotes the confidence interval of Z. The shaded grey area around each regression line represents the 95% confidence bands.
